# Mortality and Morbidity of Infants Born Extremely Preterm at Tertiary Medical Centers in China From 2010 to 2019

**DOI:** 10.1001/jamanetworkopen.2021.9382

**Published:** 2021-05-11

**Authors:** Zhicheng Zhu, Lin Yuan, Jin Wang, Qiuping Li, Chuanzhong Yang, Xirong Gao, Shangqin Chen, Shuping Han, Jiangqin Liu, Hui Wu, Shaojie Yue, Jingyun Shi, Rui Cheng, Xiuyong Cheng, Tongyan Han, Hong Jiang, Lei Bao, Chao Chen

**Affiliations:** 1 Department of Neonatology, Children’s Hospital of Fudan University, National Children’s Medical Center, Shanghai, China; 2 Department of Neonatology, Affiliated Bayi Children’s Hospital, Seventh Medical Center of Chinese People’s Liberation Army General Hospital, Beijing, China; 3 Department of Neonatology, Affiliated Shenzhen Maternity and Child Healthcare Hospital, Southern Medical University, Shenzhen, China; 4 Department of Neonatology, Hunan Children’s Hospital, Changsha, China; 5 Department of Neonatology, The Second Affiliated Hospital and Yuying Children’s Hospital of Wenzhou Medical University, Wenzhou, China; 6 Department of Neonatology, Nanjing Maternity and Child Health Care Hospital, Women’s Hospital of Nanjing Medical University, Nanjing, China; 7 Department of Neonatology, Shanghai First Maternity and Infant Hospital, Tongji University School of Medicine, Shanghai, China; 8 Department of Neonatology, First Hospital of Jilin University, Changchun, China; 9 Department of Neonatology, Xiangya Hospital, Central South University, Changsha, China; 10 Department of Neonatology, Gansu Provincial Maternity and Child Care Hospital, Lanzhou, China; 11 Department of Neonatology, Children’s Hospital of Nanjing Medical University, Nanjing, China; 12 Department of Neonatology, First Affiliated Hospital of Zhengzhou University, Zhengzhou, China; 13 Department of Neonatology, Peking University Third Hospital, Beijing, China; 14 Department of Neonatology, Affiliated Hospital of Qingdao University, Qingdao, China; 15 Department of Neonatology, Children’s Hospital of Chongqing Medical University, Chongqing, China

## Abstract

**Importance:**

Extreme prematurity is associated with a substantial burden on health care systems worldwide. However, little is known about the prognosis of infants born extremely preterm in developing countries, such as China.

**Objective:**

To describe survival and major morbidity among infants born extremely preterm in China over the past decade.

**Design, Setting, and Participants:**

This retrospective cohort study was conducted from January 1, 2010, through December 31, 2019. Included individuals were infants with gestational age less than 28 weeks discharged from 1 of 68 neonatal intensive care units located in 31 provinces in China. Data were analyzed from August through October 2020.

**Exposure:**

Extremely preterm birth.

**Main Outcomes and Measures:**

Survival to discharge and major morbidity (ie, bronchopulmonary dysplasia, grades III-IV intraventricular hemorrhage, white matter injury, stage II-III necrotizing enterocolitis, sepsis, or severe retinopathy of prematurity) were measured.

**Results:**

Among 8514 eligible infants, 5295 (62.2%) were male and 116 infants (2.0%) were small for gestational age (SGA). Overall, 5302 infants (62.3%) survived to discharge. The survival rate was 1 of 21 infants (4.8%) at 22 weeks, 13 of 71 infants (18.3%) at 23 weeks, 144 of 408 infants (35.3%) at 24 weeks, 480 of 987 infants (48.6%) at 25 weeks, 1423 of 2331 infants (61.0%) at 26 weeks, and 3241 of 4692 infants (69.1%) at 27 weeks. Survival increased from 136 of 241 infants (56.4%; 95% CI, 50.1%-62.7%) in 2010 to 1110 of 1633 infants (68.0%; 95% CI, 65.7%-70.2%) in 2019 for infants born at 24 to 27 weeks (mean difference, 11.5%; 95% CI, 4.9%-18.2%; *P* < .001), without a significant change for infants born at less than 24 weeks. Major morbidity was found in 5999 of 8281 infants overall, for a rate of 72.4%, which increased from 116 of 223 infants (52.0%; 95% CI, 45.4%-58.6%) to 1363 of 1656 infants (82.3%; 95% CI, 80.5%-84.1%) from 2010 to 2019 (mean difference, 30.3%; 95% CI, 23.5%-37.1%, *P* < .001). Regional variations in survival were identified, with an almost 2-fold increase (1.94-fold; 95% CI, 1.66-2.27; *P* < .001) from 188 of 474 infants (39.7%) in northwest China to 887 of 1153 infants (76.9%) in north China. Gestational age (adjusted risk ratio [aRR], 1.084; 95% CI, 1.063-1.105; *P* < .001), birth weight (aRR, 1.028; 95% CI, 1.020-1.036; *P* < .001), premature rupture of membranes (aRR, 1.025; 95% CI, 1.002-1.048; *P* = .03), and antenatal steroids (aRR, 1.029; 95% CI, 1.004-1.055; *P* = .02) were associated with improved survival, while being born SGA (aRR, 0.801; 95% CI, 0.679-0.945; *P* = .01), being male (aRR, 0.975; 95% CI, 0.954-0.997; *P* = .02), multiple birth (aRR, 0.955; 95% CI, 0.929-0.982; *P* = .001), having a mother with gestational diabetes (aRR, 0.946; 95% CI, 0.913-0.981; *P* = .002), and low Apgar score (aRR, 0.951; 95% CI, 0.925-0.977; *P* < .001) were found to be risk factors associated with decreased chances of survival.

**Conclusions and Relevance:**

This study found that infants born extremely preterm were at increased risk of mortality and morbidity in China, with a survival rate that improved over time and a major morbidity rate that increased. These findings suggest that more active and effective treatment strategies are needed, especially for infants born at gestational age 25 to 27 weeks.

## Key Points

**Question:**

What are the rates of mortality and major morbidity among infants born extremely preterm in China?

**Findings:**

In this multicenter cohort study across mainland China from 2010 to 2019, which included 8514 infants with gestational age less than 28 weeks, there was an improved rate of survival to discharge and an increased rate of major morbidity over the decade.

**Meaning:**

These findings suggest that infants born extremely preterm are at increased risk of mortality and morbidity in China and that more active and effective treatment strategies are needed, especially for infants born at gestational age 25 to 27 weeks.

## Teaser

This cohort study examines the association of preterm birth with risk of mortality and morbidity among infants born in China from 2010 to 2019.

## Introduction

Despite the relatively small number of infants born extremely preterm (ie, those with gestational age [GA] less than 28 weeks), accounting for 5.2% of all preterm births worldwide, they are at increased risk of mortality and morbidity.^1^ These most immature infants are at increased risk for various complications, such as bronchopulmonary dysplasia (BPD), intraventricular hemorrhage (IVH), white matter injury (WMI), necrotizing enterocolitis (NEC), sepsis, and retinopathy of prematurity (ROP), which are associated with adverse long-term outcomes.^2^ Management of such conditions in these infants poses a great challenge to families, neonatologists, and the entire health care system.^3,4^

In developed countries, large cohort studies found that the survival rate of infants born extremely preterm was 62% in England,^5^ 69% in France,^6^ 74% in Norway,^7^ and 72% in the US.^8^ Outcomes for these infants have improved over the last 2 decades.^9,10,11^ In England, the rate of survival to discharge increased from 40% in 1995 to 53% in 2006.^5^ Survival without major morbidity increased by approximately 2% per year between 1993 and 2012 in the United States.^12^ However, little is known about the prognosis of this high-risk group of infants in developing countries, such as China, which has more than 1 million preterm births annually.^13^

In China, the lower cutoff point for reporting preterm birth had long been set at 28 weeks until the latest guideline was published by the National Health Commission in 2017, removing this lower gestational threshold.^14,15,16^ With advances in perinatal and neonatal care, more and more infants have been born at earlier GA and survived.^17^ Understanding the characteristics and outcomes of infants born extremely preterm is essential for parent counseling, medical decision making, clinical management, and policy making. This study primarily aimed to evaluate mortality and major morbidity among infants born extremely preterm in China over the past decade.

## Methods

This cohort study was approved by the research ethics board of the Children’s Hospital of Fudan University with a waiver of informed consent from this ethics board because of the retrospective and observational design of the study. This study is reported following the Strengthening the Reporting of Observational Studies in Epidemiology (STROBE) reporting guideline.

### Study Design

We retrospectively studied all infants with GA less than 28 weeks discharged from 68 tertiary neonatal intensive care units (NICUs) from January 1, 2010, through December 31, 2019. These study sites are located in 31 provinces, autonomous regions, and municipalities throughout 7 geographical areas, accounting for approximately 10% of the tertiary NICUs in mainland China (eFigure 1 in the Supplement). In this newly established collaborative group, there are 35 general hospitals, 12 pediatric hospitals, and 21 maternity hospitals. All participating NICUs serve as care centers and referral centers for neonates in critical condition (including 40 NICUs at the provincial level and 28 at the municipal level), with a mean of 30 beds. They are local representatives of neonatal intensive care and have experience in multicenter clinical research. Inborn and outborn infants were included in the study. The Children’s Hospital of Fudan University, as the research center, was responsible for coordination, information integration, and data analysis.

### Data Collection

Study participants were identified by searching the electronic medical record system of each hospital based on GA and discharge year. Data collection, supervised by NICU directors, started in April 2020. Electronic medical records during initial hospitalization were reviewed by trained investigators to collect information on perinatal characteristics, neonatal diseases, and outcomes at discharge. The same diagnostic criteria were applied to all NICUs consistently over the study period. Those investigators input the data into a bespoke stand-alone database established by EpiData version 3.1 (EpiData Association) capable of logical consistency check and data verification. Meanwhile, submitted data were double checked for accuracy and completeness by the research center. All authors vouch for quality assurance.

### Definitions

Estimates of GA were obtained, in descending order of preference, from ultrasonography, last menstrual period, or New Ballard Score.^18^ Infants with a birth weight below the 10th percentile for their GA were considered small for gestational age (SGA).^19^ Elderly pregnancy was considered to have occurred in pregnant women aged older than 35 years. Maternal complications included hypertensive disorder complicating pregnancy, gestational diabetes, chorioamnionitis, placental abruption or placenta previa, and premature rupture of membranes (PROM). Hypertensive disorder complicating pregnancy and gestational diabetes were diagnosed according to national guidelines.^20,21^ Chorioamnionitis was confirmed clinically or histopathologically.^22^ We used PROM to refer to spontaneous rupture of membranes before the onset of labor. Fetal distress was diagnosed based on a rapidly deteriorating or abnormal cardiotocograph or a fetal scalp pH less than 7.2.^23^ Antenatal steroid was defined as any corticosteroid used to accelerate fetal lung maturity, regardless of doses and timing of administration. We diagnosed BPD as oxygen dependency for at least 28 days.^24^ The Papile criteria were used to grade IVH.^25^ We used WMI to refer to cystic and diffuse periventricular leukomalacia.^26^ Diagnosis of IVH and WMI was done by cranial sonography, magnetic resonance imaging, or computed tomography with the most severe findings during hospitalization. Diagnosis and grading of NEC were performed according to modified Bell criteria.^27^ Sepsis was defined by clinical symptoms and positive culture from blood or cerebrospinal fluid samples.^28,29^ Individuals who had ROP stage 3 to 5^30^ or were receiving treatment (ie, laser coagulation, intravitreal antivascular endothelial growth factor, or surgical treatment) for ROP were considered to have severe ROP.

Study main outcomes were survival to discharge and major morbidity, which was defined as the presence of BPD, IVH (grade III-IV), WMI, NEC (stage II-III), sepsis, or severe ROP. We further evaluated the proportion of deaths that occurred after withholding or withdrawing life-sustaining intensive care, including ventilation, chest compression, circulatory support, and parenteral nutrition.

### Statistical Analysis

Results were presented as mean with SD, median with interquartile range (IQR), or numbers with percentage, as appropriate. We used modified Poisson regression models with robust variance estimation to examine the association between GA and short-term outcomes, adjusting for birth weight, study site, and discharge year. Regression models assessing yearly changes in survival and morbidity rates were adjusted for GA, birth weight, and study site.

Perinatal risk factors associated with survival were evaluated using multivariable modified Poisson regression, with adjustment for the fixed effect of study sites and discharge year. Infants who died after treatments were withdrawn were excluded from the analysis. Pearson correlation coefficient and variance inflation factor were used to detect collinearity between variables. Maternal and neonatal variables, but not collinear variables, were entered into the model, including GA (in weeks), birth weight (in 100 g intervals), SGA status, male sex, elderly pregnancy status, use of in vitro fertilization (IVF), multiple pregnancy status, cesarean delivery status, maternal complications, fetal distress, 5-minute Apgar score of 7 or less, and antenatal steroid use. Data were missing for fewer than 10% of individuals in almost all measures (number of individuals with missing data ranged from 11 individuals [0.1%] for the diagnosis of BPD to 899 individuals [10.5%] for Apgar score) (Table 1 and Table 2), and values were not imputed.

**Table 1.** Maternal and Infant Characteristics by Gestational Age

Characteristic	Births, No./Total No. (%)^a^							*P* value^c^
	≤22 wk (n = 25)^b^	23 wk (n = 71)	24 wk (n = 408)	25 wk (n = 987)	26 wk (n = 2331)	27 wk (n = 4692)	Total (N = 8514)	
Infant characteristic								
Birth weight, mean (SD), g	541.8 (107.0)	603.2 (112.7)	716.0 (128.6)	827.7 (133.6)	933.6 (143.0)	1041.7 (157.7)	966.5 (180.5)	<.001
SGA	0/11	8/71 (11.3)	17/408 (4.2)	20/985 (2.0)	42/2327 (1.8)	79/4676 (1.7)	166/8478 (2.0)	<.001
Sex								
Male	18/25 (72.0)	49/71 (69.0)	252/408 (61.8)	601/987 (60.9)	1461/2331 (62.7)	2914/4692 (62.1)	5295/8514 (62.2)	<.001
Female	7/25 (28.0)	22/71 (31.0)	156/408 (38.2)	386/987 (39.1)	870/2331 (37.3)	1778/4692 (37.9)	3219/8514 (37.8)	
Fetal distress	4/24 (16.7)	5/70 (7.1)	19/399 (4.8)	49/967 (5.1)	117/2254 (5.2)	338/4528 (7.5)	532/8242 (6.5)	<.001
1-min Apgar score ≤7	24/24 (100.0)	64/70 (91.4)	307/373 (82.3)	686/903 (76.0)	1414/2066 (68.4)	2423/4179 (58.0)	4918/7615 (64.6)	<.001
5-min Apgar score ≤7	18/24 (75.0)	47/70 (67.1)	183/373 (49.1)	434/903 (48.1)	808/2066 (39.1)	1347/4179 (32.2)	2837/7615 (37.3)	<.001
Maternal characteristic								
Age, mean (SD), y	34.5 (4.5)	30.2 (4.3)	31.0 (5.1)	31.2 (5.1)	30.9 (4.9)	30.7 (5.1)	30.8 (5.1)	.009
<18	0/20	0/66	1/377 (0.3)	2/906 (0.2)	2/2099 (0.1)	16/4272 (0.4)	21/7740 (0.3)	.08
18-35	9/20 (45.0)	56/66 (84.8)	309/377 (82.0)	726/906 (80.1)	1711/2099 (81.5)	3500/4272 (81.9)	6311/7740 (81.5)	<.001
>35	11/20 (55.0)	10/66 (15.2)	67/377 (17.8)	178/906 (19.6)	386/2099 (18.4)	756/4272 (17.7)	1408/7740 (18.2)	<.001
IVF	10/25 (40.0)	23/70 (32.9)	129/398 (32.4)	326/956 (34.1)	645/2206 (29.2)	1006/4438 (22.7)	2139/8093 (26.4)	<.001
Twin or multiple pregnancy	12/25 (48.0)	36/70 (51.4)	167/400 (41.8)	415/963 (43.1)	918/2243 (40.9)	1588/4520 (35.1)	3136/8221 (38.1)	.03
Cesarean delivery	4/25 (16.0)	4/71 (5.6)	31/401 (7.7)	102/965 (10.6)	352/2257 (15.6)	1112/4545 (24.5)	1605/8264 (19.4)	<.001
PROM	7/25 (28.0)	19/71 (26.8)	103/398 (25.9)	292/960 (30.4)	746/2237 (33.3)	1539/4510 (34.1)	2706/8201 (33.0)	<.001
>18 h	7/25 (28.0)	12/70 (17.1)	74/393 (18.8)	186/948 (19.6)	472/2187 (21.6)	966/4435 (21.8)	1717/8058 (21.3)	.007
>24 h	6/25 (24.0)	8/70 (11.4)	63/393 (16.0)	158/948 (16.7)	404/2187 (18.5)	856/4435 (19.3)	1495/8058 (18.6)	<.001
Chorioamnionitis	1/24 (4.2)	1/70 (1.4)	12/393 (3.1)	17/952 (1.8)	43/2216 (1.9)	85/4455 (1.9)	159/8110 (2.0)	.09
Placental abruption or placenta previa	6/24 (25.0)	8/70 (11.4)	33/393 (8.4)	59/952 (6.2)	179/2216 (8.1)	444/4455 (10.0)	729/8110 (9.0)	.006
Hypertensive disorder complicating pregnancy	0/24	3/70 (4.3)	22/399 (5.5)	51/964 (5.3)	128/2243 (5.7)	476/4508 (10.6)	680/8208 (8.3)	<.001
Gestational diabetes	0/24	5/70 (7.1)	25/399 (6.3)	86/964 (8.9)	243/2243 (10.8)	526/4508 (11.7)	885/8208 (10.8)	<.001
Antenatal steroids	10/25 (40.0)	25/71 (35.2)	172/398 (43.2)	432/963 (44.9)	1070/2246 (47.6)	2211/4539 (48.7)	3920/8242 (47.6)	<.001

Abbreviations: IVF, in vitro fertilization; PROM, premature rupture of membranes; SGA, small for gestational age.

^a^ Denominators (ie, total No.) varied according to the number of missing data for each variable.

^b^ Data were combined into 1 category because of the small number of infants born at 21 weeks (4 infants) and 22 weeks (21 infants).

^c^ *P* values were determined for differences according to gestational age using modified Poisson regression or linear regression models, with adjustment for birth weight, study site, and discharge year. Differences in birth weight and SGA were adjusted for study site and discharge year.

**Table 2.** Survival and Major Morbidity by GA

Variable	Infants, No./Total No. (%)^a^							*P* value^c^
	≤22 wk (n = 25)^b^	23 wk (n =71)	24 wk (n = 408)	25 wk (n = 987)	26 wk (n = 2331)	27 wk (n = 4692)	Total (N = 8514)	
Survival	1/25 (4.0)	13/71 (18.3)	144/408 (35.3)	480/987 (48.6)	1423/2331 (61.0)	3241/4692 (69.1)	5302/8514 (62.3)	<.001
Active treatment	13/25 (52.0)	39/71 (54.9)	238/408 (58.3)	644/987 (65.2)	1690/2331 (72.5)	3653/4692 (77.9)	6277/8514 (73.7)	<.001
Death								
Despite active treatment	12/25 (48.0)	26/71 (36.6)	94/408 (23.0)	164/987 (16.6)	267/2331 (11.5)	412/4692 (8.8)	975/8514 (11.5)	<.001
After withdrawing treatment	12/25 (48.0)	32/71 (45.1)	170/408 (41.7)	343/987 (34.8)	641/2331 (27.5)	1039/4692 (22.1)	2237/8514 (26.3)	<.001
Postnatal time until death, d (n = 3073)								
<1	6/23 (26.1)	10/56 (17.9)	40/253 (15.8)	77/490 (15.7)	125/867 (14.4)	156/1384 (11.3)	414/3073 (13.5)	.17
1-3	9/23 (39.1)	30/56 (53.6)	104/253 (41.1)	192/490 (39.2)	294/867 (33.9)	474/1384 (34.2)	1103/3073 (35.9)	.02
4-7	3/23 (13.0)	8/56 (14.3)	42/253 (16.6)	66/490 (13.5)	106/867 (12.2)	202/1384 (14.6)	427/3073 (13.9)	.79
8-28	3/23 (13.0)	5/56 (8.9)	41/253 (16.2)	101/490 (20.6)	194/867 (22.4)	295/1384 (21.3)	639/3073 (20.8)	.47
>28	2/23 (8.7)	3/56 (5.4)	26/253 (10.3)	54/490 (11.0)	148/867 (17.1)	257/1384 (18.6)	490/3073 (15.9)	<.001
Length of stay for infants who survived, median (IQR), d	NA^d^	121.5 (74-151)	98 (56-123)	85 (60-104)	76 (58-89)	64 (51-78)	68 (53-85)	<.001
Corrected GA at discharge for survivors, mean (SD), wk	NA^d^	39.0 (7.6)	38.7 (7.1)	38.3 (5.6)	37.7 (4.4)	37.4 (3.9)	37.6 (4.3)	<.001
Cranial imaging	19/25 (76.0)	65/71 (91.5)	351/408 (86.0)	869/987 (88.0)	2074/2331 (89.0)	4188/4692 (89.3)	7566/8514 (88.9)	.04
ROP screening	2/25 (8.0)	12/71 (16.9)	131/402 (32.6)	442/969 (45.6)	1285/2262 (56.8)	2802/4552 (61.6)	4674/8281 (56.4)	<.001
Major morbidity								
BPD^e^	3/3 (100.0)	12/13 (92.3)	127/147 (86.4)	406/485 (83.7)	1099/1385 (79.4)	2188/3146 (69.5)	3835/5179 (74.0)	.06
IVH grade III-IV^f^	5/19 (26.3)	16/65 (24.6)	89/340 (26.2)	152/845 (18.0)	331/2005 (16.5)	418/4088 (10.2)	1011/7362 (13.7)	<.001
WMI^f^	2/19 (10.5)	10/65 (15.4)	32/351 (9.1)	135/869 (15.5)	353/2074 (17.0)	730/4188 (17.4)	1262/7566 (16.7)	.16
NEC stage II-III	2/25 (8.0)	1/70 (1.4)	24/395 (6.1)	64/947 (6.8)	136/2216 (6.1)	304/4493 (6.8)	531/8146 (6.5)	.80
Sepsis	7/25 (28.0)	24/71 (33.8)	133/402 (33.1)	368/967 (38.1)	840/2257 (37.2)	1624/4537 (35.8)	2996/8259 (36.3)	.13
Severe ROP^g^	1/2 (50.0)	5/12 (41.7)	63/128 (49.2)	137/425 (32.2)	284/1243 (22.8)	358/2708 (13.2)	848/4518 (18.8)	<.001
Any major morbidity	11/25 (44.0)	36/71 (50.7)	261/402 (64.9)	675/969 (69.7)	1690/2262 (74.7)	3326/4552 (73.1)	5999/8281 (72.4)	<.001
Survival without major morbidity^h^	0/25	2/71 (2.8)	14/402 (3.5)	46/969 (4.7)	171/2262 (7.6)	547/4552 (12.0)	780/8281 (9.4)	<.001

Abbreviations: BPD, bronchopulmonary dysplasia; GA, gestational age; IQR, interquartile range; IVH, intraventricular hemorrhage; NA, not applicable; NEC, necrotizing enterocolitis; ROP, retinopathy of prematurity; WMI, white matter injury.

^a^ Denominators (ie, total No.) varied according to the number of missing data for each variable.

^b^ Data were combined into 1 category because of the small number of infants born at 21 weeks (4 infants) and 22 weeks (21 infants).

^c^ *P* values were determined for differences by GA using modified Poisson regression or linear regression models, with adjustment for birth weight, study site, and discharge year. Differences in length of stay were analyzed by Spearman rank correlation test.

^d^ Among infants born at 22 weeks or earlier, 1 infant survived, with a length of stay of 95 days and a corrected GA at discharge of 35.9 weeks.

^e^ Proportions are among infants who survived more than 28 days. Total denominator includes 4700 infants who survived with obtainable postnatal days until discharge at later than 28 days and 490 infants who died after 28 postnatal days, excluding 11 infants for whom diagnosis of BPD was unknown.

^f^ Total proportion is among 7566 infants who underwent cranial imaging (ie, cranial sonography, magnetic resonance imaging, or computed tomography), excluding 204 infants for IVH grade for whom grade was unknown.

^g^ Total proportion is among 4674 infants who received ROP examination, excluding 156 infants for whom the stage or treatments of ROP were not available.

^h^ Total proportion is among the overall study population, excluding 233 infants for whom diagnosis of major morbidity was unknown.

*P* values were 2-tailed, and *P* < .05 was considered statistically significant. Data analyses were performed using SPSS statistical software version 24.0 (IBM) from August through October 2020.

## Results

### Study Population

We identified 8514 eligible infants born at 21 weeks and 4 daysto 27 weeks and 6 days of gestation with a mean (SD) birth weight of 967 (181) g, of whom 5295 (62.2%) were male and 116 individuals (2.0%) were SGA. Over the 10-year period, there were more infants in the lower GA categories, with the proportion of infants with GA less than 26 weeks increasing from 33 of 241 infants (13.7%; 95% CI, 9.3%-18.1%) in 2010 to 323 of 1656 infants (19.5%; 95% CI, 17.6%-21.4%) in 2019. Moreover, the proportion of enrolled infants out of all infants discharged from NICUs, which was calculated excluding infants from hospitals without available data for total number of discharged infants in the corresponding study year, increased from 158 of 107 985 infants in 2010 (0.15%) to 1398 of 201 877 infants in 2019 (0.69%). (eTable 1 in the Supplement).

### Perinatal Characteristics

Demographic and perinatal characteristics by GA and discharge year are presented in Table 1 and eTable 1 in the Supplement, respectively. Proportions are given out of the total number of individuals with data for that characteristic. Low Apgar score was more common for infants with the lowest GA, and the rate decreased over time for all infants. Elderly pregnancy was found in 1408 of 7740 mothers (18.2%), and IVF was used by 2139 of 8093 of mothers (26.4%). Overall, 5085 of 8221 births (61.9%) were singleton and 1605 of 8264 infants (19.4%) were cesarean deliveries. From 2010 to 2019, the proportion of births that were from elderly pregnancies increased from 25 of 209 individuals (12.0%; 95% CI, 7.5%-16.4%) to 296 of 1621 individuals (18.3%; 95% CI, 16.4%-20.1%), the proportion from IVF increased from 42 of 218 individuals (19.3%; 95% CI, 14.0%-24.5%) to 428 of 1617 individuals (26.5%; 95% CI, 24.3%-28.6%), and the proportion that were cesarean deliveries increased from 52 of 223 individuals (23.3%; 95% CI, 17.7%-28.9%) to 416 of 1648 individuals (25.2%; 95% CI, 23.1%-27.3%).

Maternal complications included 2706 of 8201 mothers with PROM (33.0%), 159 of 8110 mothers with chorioamnionitis (2.0%), 729 of 8110 mothers with placental abruption or placenta previa (9.0%), 680 of 8208 mothers with hypertensive disorder complicating pregnancy (8.3%), and 885 of 8208 mothers with gestational diabetes (10.8%). A rising trend was found for all these complications except hypertensive disorder complicating pregnancy from 2010 to 2019.

The administration rate of antenatal steroids ranged from 10 of 25 mothers (40.0%) at 21 to 22 weeks to 2211 of 4539 mothers (48.7%) at 27 weeks and increased from 54 of 233 mothers (24.2%; 95% CI, 18.5%-29.9%) in 2010 to 957 of 1650 mothers (58.0%; 95% CI, 55.6%-60.4%) in 2019. Regional variations existed, with the highest use rate in the south (639 of 1060 mothers [60.3%]) and the lowest in the northeast (100 of 372 mothers [26.9%]) (eTable 2 in the Supplement).

### Survival

Overall, 5302 of 8514 infants survived to discharge, for a rate of 62.3% (95% CI, 61.2%-63.3%). Among 6277 infants receiving ongoing active treatment, 975 infants (15.5%) died, and among 2237 infants for whom a decision was made to limit or withdraw life-sustaining care, all infants died. Among all deaths, 414 deaths (13.5%) occurred within the first day of life and 1944 deaths (63.3%) occurred in the first week. The median (IQR) length of stay for infants who survived was 68 (53-85) days, increasing from 58 (22-81) days in 2010 to 72 (58-88) days in 2019 (eTable 3 in the Supplement).

Survival to discharge increased with increasing GA: 1 of 21 infants (4.8%) at 22 weeks, 13 of 71 infants (18.3%) at 23 weeks, 144 of 408 infants (35.3%) at 24 weeks, 480 of 987 infants (48.6%) at 25 weeks, 1423 of 2331 infants (61.0%) at 26 weeks, and 3241 of 4692 infants (69.1%) at 27 weeks (Table 2). There were 4 infants born at 21 weeks, and none of them survived to discharge.

**Figure 1.**
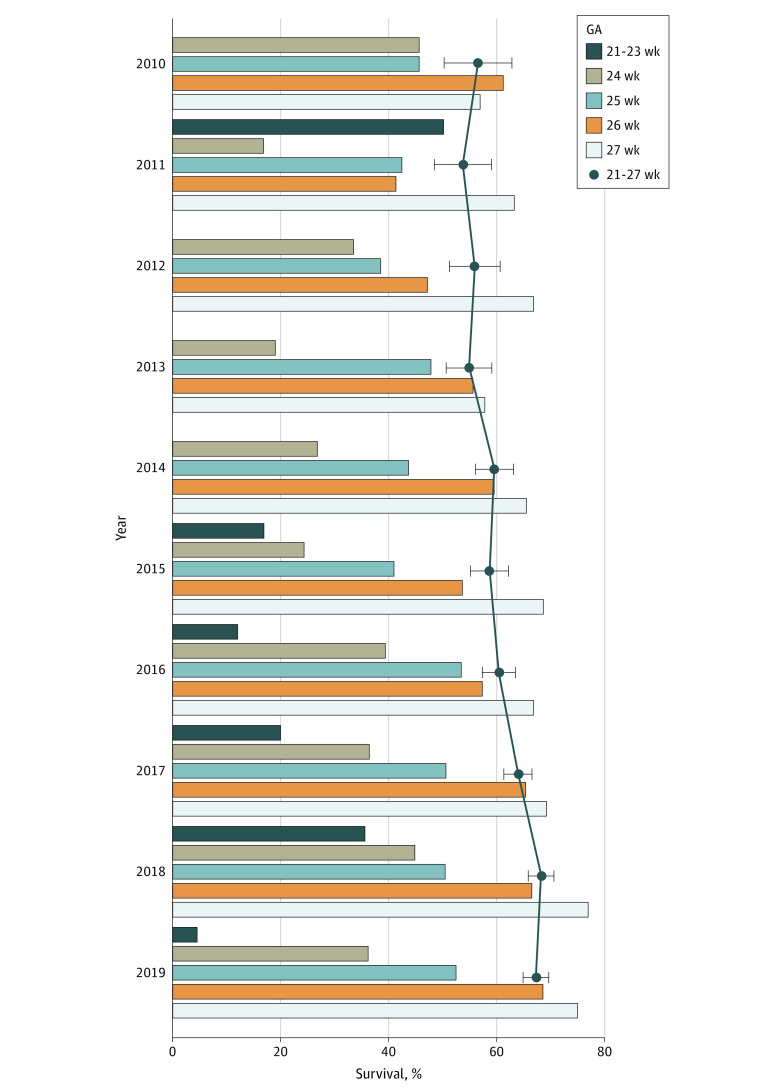
Survival to Discharge by Gestational Age (GA) Polyline indicates survival rate of infants born extremely preterm; whiskers, 95% CI; columns, analysis stratified by GA. The survival rate, adjusted for GA, birth weight, and study site improved over time: adjusted risk ratio for the change per year, 1.053; 95% CI, 1.046-1.060; *P* < .001. The total number of infants in each GA category is listed in Table 1.

The survival rate improved from 136 of 241 infants (56.4%; 95% CI, 50.1%-62.7%) in 2010 to 1111 of 1656 infants (67.1%; 95% CI, 64.8%-69.4%) in 2019 (mean difference, 10.7%; 95% CI, 4.0%-17.3%; *P* < .001). The trend in survival varied by GA, as shown in Figure 1 and eTable 3 in the Supplement. There was a significant increase in survival rate for infants born at GA 24 to 27 weeks, from 136 of 241 infants (56.4%; 95% CI, 50.1%-62.7%) in 2010 to 1110 of 1633 infants (68.0%; 95% CI, 65.7%-70.2%) in 2019 (mean difference, 11.5%; 95% CI, 4.9%-18.2%; *P* < .001). However, no significant change was found for infants born at GA less than 24 weeks.

Moreover, the survival rate ranged from 3 of 21 infants (14.3%) to 652 of 740 infants (88.1%) among study sites. Outcomes for infants from different regions are shown in eTable 4 and eFigure 2 in the Supplement. Significant differences in survival were identified, with an almost 2-fold increase (1.94-fold; 95% CI, 1.66-2.27; *P* < .001) from 188 of 474 infants (39.7%) in the northwest to 887 of 1153 infants (76.9%) in north China. Improved survival was observed in all regions except northwest and north China (eTable 5 in the Supplement).

### Major Neonatal Morbidity

Among 5179 infants, BPD occurred in 3835 infants (74.0%); the condition was evaluated in infants who survived more than 28 days. Among 7566 infants examined by cranial imaging, 1262 infants (16.7%) had WMI and 3691 infants (48.8%) developed IVH. Among 8219 infants, NEC occurred in 830 infants (10.1%); of these, 531 infants were diagnosed with stage II to III NEC and 57 developed post-NEC strictures. A total of 2996 of 8259 infants (36.3%) developed sepsis. Ophthalmologic examinations were performed in 4674 of 8281 infants (56.4%), and 3060 of 4674 infants (65.5%) met the diagnostic criteria of ROP. Among 4518 infants, 848 infants (18.8%) had severe ROP.

**Figure 2.**
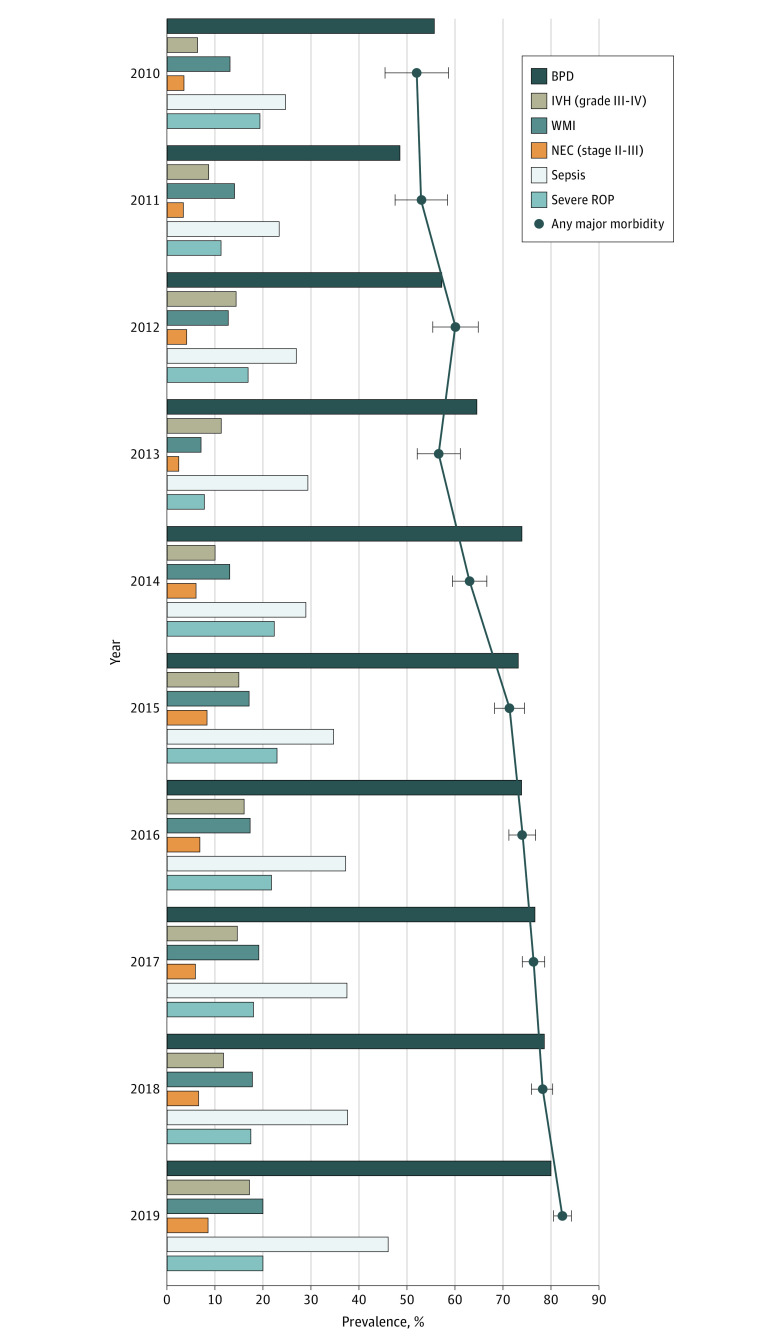
Major Morbidity Prevalence BPD indicates bronchopulmonary dysplasia; IVH, intraventricular hemorrhage; NEC, necrotizing enterocolitis; ROP, retinopathy of prematurity; WMI, white matter injury; columns, prevalence of major neonatal complications; major morbidity, presence of BPD, IVH grade III to IV, WMI, NEC stage II to III, sepsis, or severe ROP; polyline, trend in major morbidity; whiskers, 95% CI. Risk ratio adjusted for gestational age, birth weight, and study site for the change per year, 1.055; 95% CI, 1.048-1.061; *P* < .001.

As shown in Table 2, the prevalence of IVH grade III to IV and severe ROP were inversely associated with GA. The prevalence of IVH was 5 of 19 infants (26.3%) at GA 21 to 22 weeks and 418 of 4088 infants at GA 27 weeks (*P* < .001); the prevalence of severe ROP was 1 of 2 infants (50.0%) at GA 21 to 22 weeks and 358 of 2708 infants (13.2%) at 27 weeks (*P* < .001). The prevalence increased from 2010 to 2019 for BPD (64 of 115 infants [55.7%] vs 954 of 1194 infants [79.9%]; *P* < .001), IVH grade III to IV (14 of 219 infants [6.4%] vs 248 of 1444 infants [17.2%]; *P* < .001), WMI (29 of 222 infants [13.1%] vs 299 of 1492 infants [20.0%]; *P* = .001), NEC stage II to III (8 of 222 infants [3.6%] vs 136 of 1572 infants [8.7%]; *P* < .001), and sepsis (55 of 223 infants [24.7%] vs 762 of 1656 infants [46.0%]; *P* < .001), but the prevalence of severe ROP did not change significantly despite increasing rates of ROP screening (Figure 2; eTable 3 in the Supplement).

Overall, major morbidity was present in 5999 of 8281 infants (72.4%), and 780 infants (9.4%) survived without any major morbidity. Major morbidity increased from 11 of 25 infants (44.0%) at 21 to 22 weeks to 3326 of 4552 infants (73.1%) at 27 weeks (Table 2). Additionally, there was an increasing trend for major morbidity over time, from 116 of 223 infants (52.0%; 95% CI, 45.4%-58.6%) in 2010 to 1363 of 1656 infants (82.3%; 95% CI, 80.5%-84.1%) in 2019 (mean difference, 30.3%; 95% CI, 23.5%-37.1%; *P* < .001).(eTable 3 in the Supplement). Meanwhile, the survival rate without major morbidity decreased from 48 infants (21.5%; 95% CI, 16.1%-27.0%) in 2010 to 92 infants (5.6%; 95% CI, 4.5%-6.7%) in 2019 (*P* < .001).

### Perinatal Risk Factors Associated With Survival

In multivariate analysis of infants with active treatments, we found that GA (adjusted risk ratio [aRR], 1.084; 95% CI, 1.063-1.105; *P* < .001), birth weight (aRR, 1.028; 95% CI, 1.020-1.036; *P* < .001), PROM (aRR, 1.025; 95% CI, 1.002-1.048; *P* = 0.03), and antenatal steroids (aRR, 1.029; 95% CI, 1.004-1.055; *P* = .02) were associated with improved survival. However, being born SGA (aRR, 0.801; 95% CI, 0.679-0.945; *P* = .01), being male (aRR, 0.975; 95% CI, 0.954-0.997; *P* = .02), multiple birth (aRR, 0.955; 95% CI, 0.929-0.982; *P* = .001), having a mother with gestational diabetes (aRR, 0.946; 95% CI, 0.913-0.981; *P* = .002), and low Apgar score (aRR, 0.951; 95% CI, 0.925-0.977; *P* < .001) were found to be risk factors associated with decreased chance of survival (Table 3). Comparison of perinatal characteristics between infants who survived and those who died and multivariate analysis in the overall study group are shown in eTable 6 and eTable 7 in the Supplement.

**Table 3.** Multivariable Regression Analysis of Perinatal Risk Factors Associated With Survival Among Infants Receiving Active Treatment

Variable	Crude model		Adjusted model^a^	
	RR (95% CI)	*P* value	aRR (95% CI)	*P* value
GA, wk	1.080 (1.058-1.102)	<.001	1.084 (1.063-1.105)	<.001
Birth weight group (100 g intervals)	1.025 (1.017-1.033)	<.001	1.028 (1.020-1.036)	<.001
SGA	0.796 (0.666-0.952)	.01	0.801 (0.679-0.945)	.01
Male	0.967 (0.945-0.990)	.004	0.975 (0.954-0.997)	.02
Fetal distress	1.000 (0.952-1.050)	.99	1.014 (0.965-1.066)	.58
5-min Apgar score ≤7	0.929 (0.904-0.955)	<.001	0.951 (0.925-0.977)	<.001
Elderly pregnancy	1.024 (0.997-1.052)	.08	1.005 (0.979-1.031)	.72
IVF	1.010 (0.980-1.042)	.52	1.013 (0.983-1.043)	.41
Twin or multiple pregnancy	0.935 (0.908-0.963)	<.001	0.955 (0.929-0.982)	.001
Cesarean delivery	1.012 (0.984-1.041)	.39	0.985 (0.958-1.013)	.30
PROM	1.023 (1.000-1.047)	.05	1.025 (1.002-1.048)	.03
Chorioamnionitis	1.004 (0.930-1.084)	.92	0.999 (0.926-1.077)	.97
Placental abruption or placenta previa	0.994 (0.955-1.034)	.75	0.996 (0.959-1.035)	.83
Hypertensive disorder complicating pregnancy	0.988 (0.945-1.032)	.59	0.991 (0.951-1.033)	.67
Gestational diabetes	0.957 (0.923-0.993)	.02	0.946 (0.913-0.981)	.002
Antenatal steroids	1.076 (1.050-1.102)	<.001	1.029 (1.004-1.055)	.02

Abbreviations: aRR, adjusted risk ratio; GA, gestational age; IVF, in vitro fertilization; PROM, premature rupture of membranes; RR, risk ratio; SGA, small for gestational age.

^a^ Analysis was performed with adjustment for study site and discharge year.

## Discussion

China, a populous country, ranked second globally in the number of preterm births.^1^ Along with social progress, economic development, and medical improvement, the total number of infants born extremely preterm and its proportion among all discharged infants increased steadily over time. Care of these infants has become a critical and urgent issue in China. This cohort study provides valid and detailed information on the short-term outcomes of infants born extremely preterm in mainland China. We found that these infants were at increased risk of mortality and morbidity and that survival rate improved and major morbidity rate increased over time. The strengths of this study are a large number of infants from a nationwide cohort and a long study period spanning 10 years.

This study found that several factors were associated with short-term outcomes after extremely preterm birth. We found that survival increased with advancing GA, which was consistent with the results of a 2010 study.^8^ Researchers have reported improved survival over time for infants born extremely preterm in Canada,^31^ the US,^12^ and the Republic of Korea.^32^ Similarly, we found a significant increase in survival from 2010 to 2019 for infants born at 24 to 27 weeks, but we did not find an increase for infants born earlier than 24 weeks. A study from England^5^ found that survival increased from 40% in 1997 to 53% in 2006 overall, but without significant improvement for infants born at 22 to 23 weeks. Such variations in trend according to GA may be associated with differences in therapeutic difficulty among infants in different GA groups and decreased willingness to provide active obstetrical and neonatal care for infants at the lowest GA. It could also be a reflection of the biological limit of viability, which cannot be extended easily by medical advances.

As other studies found,^33,34^ we found that survival for infants born extremely preterm varied among hospitals and regions. A study from Europe^35^ found that more than four-fifths of regional variation could not be explained by maternal, pregnancy, or infant characteristics. China has a vast territory with inequities in economic development, medical technology, and quality of health care provision. We found that the lowest survival rate was in the northwest region, which has a subaverage disposable income level per capita for China. Regional disparities in the resources of neonatal medicine, such as experienced professionals and advanced equipment, may be associated with clear differences in outcomes.^36^ Additionally, such regional variations may be ascribed to different care practices among units and regions owing to a lack of consensus on clinical practices; for example, we found a divergence in use of active treatments, prenatal corticosteroids,^37^ antibiotics,^38^ and human milk feeding.^39^

We identified multiple favorable and adverse factors associated with survival, which are largely in keeping with results from 2 previous studies,^40,41^ while 2 other studies found different results. Blumenfeld et al^42^ reported no differences in neonatal mortality between those born with and without PROM. Persson et al^43^ found that maternal diabetes was not associated with increased mortality in high-income countries. However, these studies were performed without considering active treatment. Furthermore, because of the retrospective design of our study and missing data for obstetric interventions, such as maternal antibiotic and tocolysis, associations between these perinatal factors and outcomes should be interpreted with caution.

In line with national trend reports, we observed a significant increase in elderly pregnancy over the study period, which might be associated with the announcement of universal 2-child policy in October 2015.^44^ Notably, although the number of infants receiving antenatal steroids has increased over the last decade, the overall administration rate in China was still much lower than in developed countries.^12^ The present guideline from the Chinese Obstetrics and Gynecology Society recommends use of antenatal corticosteroids between 28 and 34 weeks of gestation without mentioning women who are at risk of preterm delivery at less than 28 weeks’ gestation.^14^ Practices for extremely preterm birth have not been unified, and large variations were found across different sites and regions. Therefore, it is necessary to reinforce and standardize prenatal management.

We are encouraged by the improved survival of infants born extremely preterm over time, but there are still grounds for concern. First, our study found a high proportion of infants who died after a decision was made to limit or withdraw life-sustaining care. Neonatologists strive to rescue infants with a reasonable likelihood of an acceptable long-term quality of life, but decisions for active treatment, in fact, are complex in clinical scenarios and remain controversial worldwide. In developed countries, there was general agreement to provide comfort care at 22 weeks and active care at 25 weeks, while a wide variation in recommendations was seen for infants with GA from 23 to 24 weeks.^45^ In China, the decision to withhold or withdraw care was ethically optional and made primarily by parents. A survey found that the main reasons for parental decision to withdraw care were economic burden and fear of poor or uncertain outcomes.^17^ Our study could encourage health care professionals and parents to reassess their attitudes toward care at extremely low GA. More active and effective treatment strategies are needed, especially for infants born at later than GA 25 weeks. Certainly, medical decisions should be made in the best interest of each individual by a team discussion among obstetricians, neonatologists, and families, considering not only GA, but also clinical conditions, socioeconomic issues, and other important factors associated with prognosis.

Second, infants born extremely preterm are still at increased risk of morbidity. In our study, 9.4% of infants survived to discharge without any major morbidity, and the morbidity rate increased over time. By contrast, the rates of NEC, BPD, periventricular leukomalacia, and severe ROP decreased from 1997 to 2011 in France, with an increasing proportion of infants surviving without severe morbidity.^6^ The observed increase in morbidity in our study may be associated with the higher survival rate of infants with the most premature births. Furthermore, this increase may be associated with a change in practices and advances in diagnostic technology over time, such as improved quality of neuroimaging. Because these major complications have all been associated with neurodevelopmental impairments,^46^ we suspect that high rates of long-term disorders may exist in infants who survived, calling for continued interventions and updated clinical practice guidelines to improve the outcomes.

### Limitations

This study has several limitations. First, our results are not population based. The mortality rate may be underestimated because infants who died in the delivery room were not included, and failure of access to NICUs still exists owing to imperfect transportation systems, especially in less developed regions.^47^ Second, participating hospitals were not selected randomly, and all were large tertiary centers in urban areas, which may limit generalizability. Third, data were retrospectively collected and long-term outcomes were not available.

## Conclusions

This study found that infants born extremely preterm in China were at high risk of mortality and morbidity. Although we found that the survival rate improved over time, major morbidity increased. These findings suggest that more active and effective treatment strategies are needed, especially for infants born at GA 25 to 27 weeks.

## Notes

Supplement. **eFigure 1.** Geographical Distribution of Participating Hospitals and Enrolled Infants **eFigure 2.** Survival to Discharge of Infants Born Extremely Preterm by Region **eTable 1.** Maternal and Infant Characteristics for Extremely Preterm Births From 2010 to 2019 **eTable 2.** Maternal and Infant Characteristics for Extremely Preterm Births by Region **eTable 3.** Survival and Major Morbidity of Infants Born Extremely Preterm From 2010 to 2019 **eTable 4.** Survival and Major Morbidity of Infants Born Extremely Preterm by Region **eTable 5.** Survival and Major Morbidity of Infants Born Extremely Preterm by Region From 2010 to 2019 **eTable 6.** Perinatal and Maternal Characteristics for Infants Who Survived vs Did Not Survive **eTable 7.** Multivariable Regression Analysis of Perinatal Risk Factors for Survival in Total Study Group
