# Supplementary material for: Mortality and Morbidity of Infants Born Extremely Preterm at Tertiary Medical Centers in China From 2010 to 2019
Source: JAMA Netw Open. 2021 May 11;4(5):e219382. doi: 10.1001/jamanetworkopen.2021.9382 (PMC8114138; doi:10.1001/jamanetworkopen.2021.9382)
Supplement: Supplement. — eFigure 1. Geographical Distribution of Participating Hospitals and Enrolled Infants eFigure 2. Survival to Discharge of Infants Born Extremely Preterm by Region eTable 1. Maternal and Infant Characteristics for Extremely Preterm Births From 2010 to 2019 eTable 2. Maternal and Infant Characteristics for Extremely Preterm Births by Region eTable 3. Survival and Major Morbidity of Infants Born Extremely Preterm From 2010 to 2019 eTable 4. Survival and Major Morbidity of Infants Born Extremely Preterm by Region eTable 5. Survival and Major Morbidity of Infants Born Extremely Preterm by Region From 2010 to 2019 eTable 6. Perinatal and Maternal Characteristics for Infants Who Survived vs Did Not Survive eTable 7. Multivariable Regression Analysis of Perinatal Risk Factors for Survival in Total Study Group [file jamanetwopen-e219382-s001.pdf]

Supplemental Online Content

Zhu Z, Yuan L, Wang J, et al. Mortality and morbidity of infants born extremely preterm at tertiary medical centers in China from 2010 to 2019. *JAMA Netw Open*. 2021;4(5):e219382. doi:10.1001/jamanetworkopen.2021.9382

eFigure 1. Geographical Distribution of Participating Hospitals and Enrolled Infants
eFigure 2. Survival to Discharge of Infants Born Extremely Preterm by Region
eTable 1. Maternal and Infant Characteristics for Extremely Preterm Births From 2010 to 2019
eTable 2. Maternal and Infant Characteristics for Extremely Preterm Births by Region
eTable 3. Survival and Major Morbidity of Infants Born Extremely Preterm From 2010 to 2019
eTable 4. Survival and Major Morbidity of Infants Born Extremely Preterm by Region
eTable 5. Survival and Major Morbidity of Infants Born Extremely Preterm by Region From 2010 to 2019
eTable 6. Perinatal and Maternal Characteristics for Infants Who Survived vs Did Not Survive
eTable 7. Multivariable Regression Analysis of Perinatal Risk Factors for Survival in Total Study Group

This supplemental material has been provided by the authors to give readers additional information about their work.

© 2021 Zhu Z et al. *JAMA Network Open.*

eFigure 1. Geographical Distribution of Participating Hospitals and Enrolled Infants

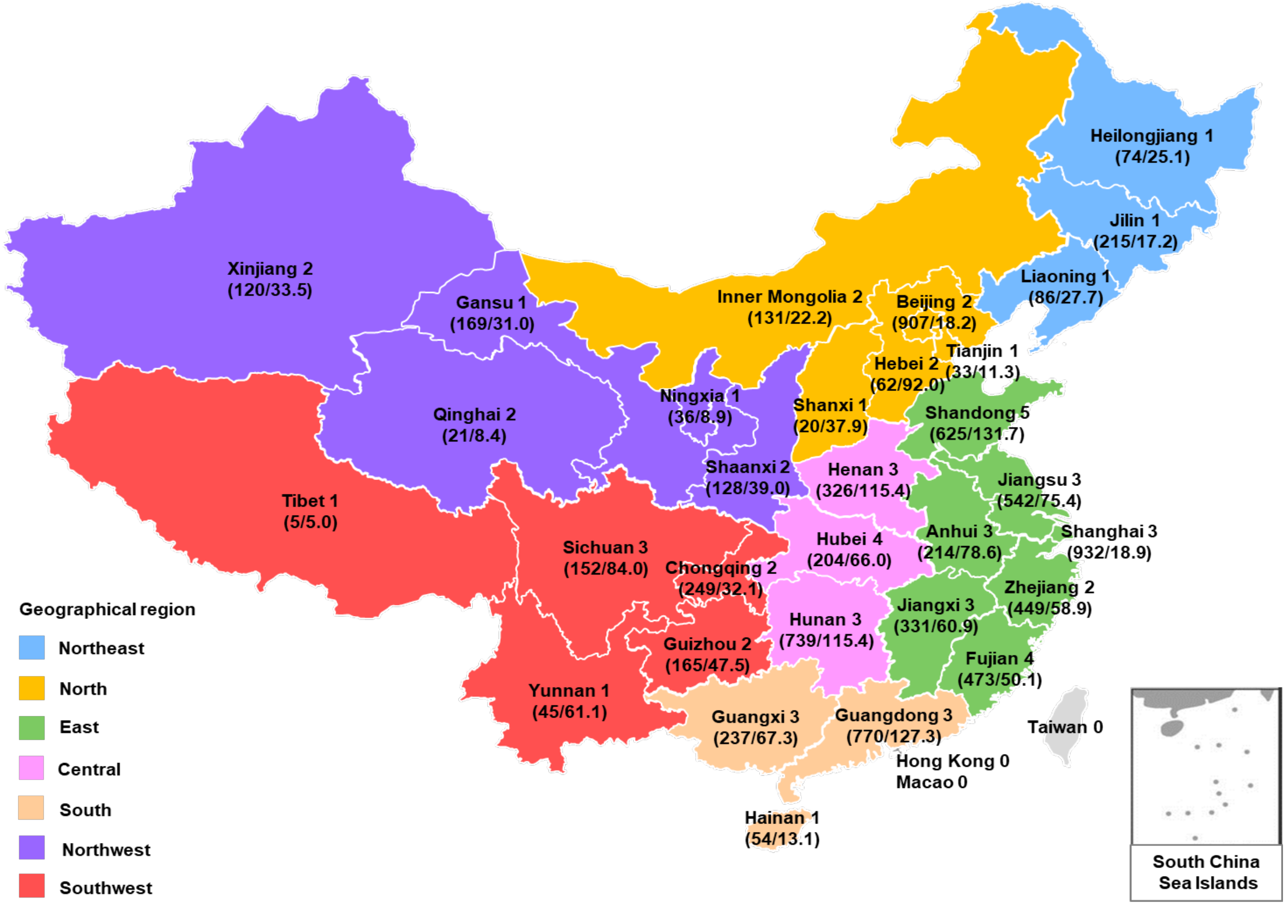

The name of each province, autonomous region, or municipality followed by the number of study sites was presented on the map of China. Number of enrolled infants/average birth population (10000 persons) was shown in the brackets. The average birth population between 2010-2019 in each province was calculated based on data released by National Bureau of Statistics of China. Different color represents different geographical regions.

© 2021 Zhu Z et al. *JAMA Network Open.*

eFigure 2. Survival to Discharge of Infants Born Extremely Preterm by Region

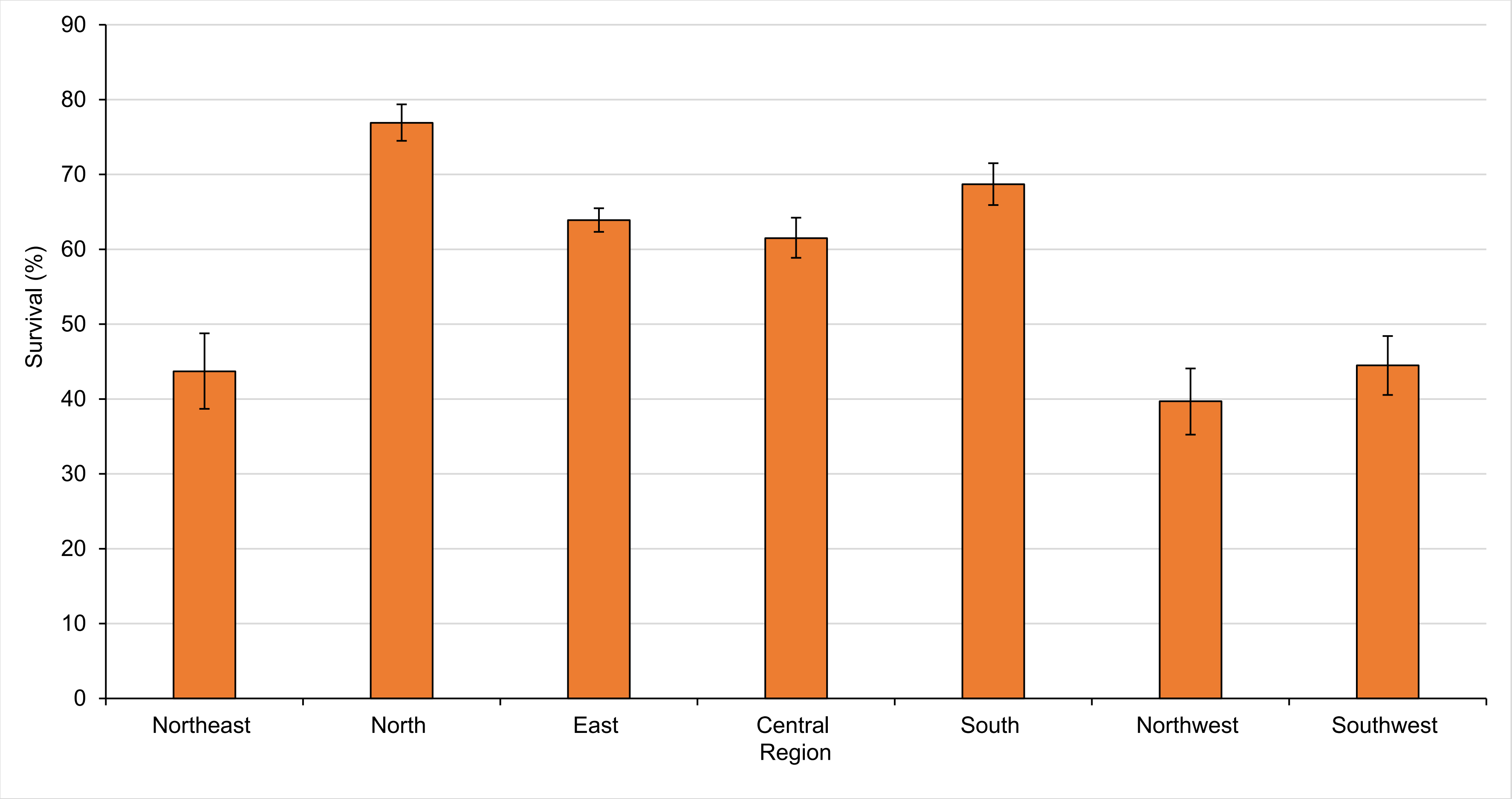

For each geographical region, percentages of extremely preterm infants who survived to discharge were presented. Error bars indicate 95% CI.

© 2021 Zhu Z et al. *JAMA Network Open.*

eTable 1. Maternal and Infant Characteristics for Extremely Preterm Births From 2010 to 2019[a]

**Characteristics**	**2010 (N=241)**	**2011 (N=347)**	**2012 (N=439)**	**2013 (N=537)**	**2014 (N=752)**	**2015 (N=828)**	**2016 (N=968)**	**2017 (N=1293)**	**2018 (N=1453)**	**2019 (N=1656)**	**Total (N=8514)**	***P* value[b]**
Infant characteristics												
GA, mean (SD), wk	26.8 (0.9)	26.8 (0.9)	26.7 (1.0)	26.9 (0.9)	26.8 (1.0)	26.7 (1.0)	26.7 (1.0)	26.7 (1.0)	26.7 (1.0)	26.7 (1.0)	26.7 (1.0)	0.31
BW, mean (SD), g	1009.8 (199.8)	1007.1 (195.5)	983.9 (189.3)	989.1 (171.9)	984.9 (179.0)	970.3 (172.5)	967.4 (180.8)	961.7 (173.9)	956.1 (180.9)	941.6 (179.5)	966.5 (180.5)	<0.001
SGA	4/241 (1.7)	5/347 (1.4)	2/437 (0.5)	7/536 (1.3)	12/749 (1.6)	15/826 (1.8)	23/964 (2.4)	23/1285 (1.8)	32/1448 (2.2)	43/1645 (2.6)	166/8478 (2.0)	0.86
Male	156/241 (64.7)	232/347 (66.9)	298/439 (67.9)	331/537 (61.6)	497/752 (66.1)	500/828 (60.4)	600/968 (62.0)	818/1293 (63.3)	885/1453 (60.9)	978/1656 (59.1)	5295/8514 (62.2)	0.10
Fetal distress	15/223 (6.7)	21/325 (6.5)	30/416 (7.2)	39/477 (8.2)	45/703 (6.4)	44/767 (5.7)	39/968 (4.0)	96/1275 (7.5)	90/1432 (6.3)	113/1656 (6.8)	532/8242 (6.5)	0.01
1-min Apgar score≤7	144/199 (72.4)	191/292 (65.4)	246/364 (67.6)	271/420 (64.5)	396/626 (63.3)	460/700 (65.7)	586/878 (66.7)	769/1199 (64.1)	875/1386 (63.1)	980/1551 (63.2)	4918/7615 (64.6)	<0.001
5-min Apgar score≤7	83/199 (41.7)	111/292 (38.0)	150/364 (41.2)	158/420 (37.6)	253/626 (40.4)	282/700 (40.3)	338/878 (38.5)	421/1199 (35.1)	488/1386 (35.2)	553/1551 (35.7)	2837/7615 (37.3)	<0.001
Proportion in all discharged infants, % (n/N)[c]	0.15 (158/ 107985)	0.20 (246/ 121372)	0.23 (326/ 142501)	0.26 (391/ 150930)	0.33 (568/ 174376)	0.45 (790/ 175956)	0.46 (852/ 186769)	0.58 (1119/ 192627)	0.63 (1239/ 197162)	0.69 (1398/ 201877)	0.43 (7087/ 1651555)	<0.001
Maternal characteristics												
Maternal age, mean (SD), y	29.6 (5.2)	29.6 (5.1)	30.2 (5.1)	30.1 (5.5)	30.2 (5.1)	30.7 (4.6)	30.8 (5.0)	31.5 (5.2)	31.2 (5.0)	31.1 (5.0)	30.8 (5.1)	<0.001
<18y	1/209 (0.5)	1/293 (0.3)	1/392 (0.3)	1/436 (0.2)	2/651 (0.3)	1/740 (0.1)	3/893 (0.3)	0/1177	3/1328 (0.2)	8/1621 (0.5)	21/7740 (0.3)	0.27
18-35y	183/209 (87.6)	246/293 (84.0)	330/392 (84.2)	368/436 (84.4)	545/651 (83.7)	615/740 (83.1)	733/893 (82.1)	909/1177 (77.2)	1065/132 8 (80.2)	1317/162 1 (81.2)	6311/7740 (81.5)	<0.001

© 2021 Zhu Z et al. *JAMA Network Open.*

Characteristics	2010 (N=241)	2011 (N=347)	2012 (N=439)	2013 (N=537)	2014 (N=752)	2015 (N=828)	2016 (N=968)	2017 (N=1293)	2018 (N=1453)	2019 (N=1656)	Total (N=8514)	*P* value[b]
>35y	25/209 (12.0)	46/293 (15.7)	61/392 (15.6)	67/436 (15.4)	104/651 (16.0)	124/740 (16.8)	157/893 (17.6)	268/1177 (22.8)	260/1328 (19.6)	296/1621 (18.3)	1408/7740 (18.2)	<0.001
In vitro fertilization	42/218 (19.3)	48/319 (15.0)	96/408 (23.5)	113/472 (23.9)	156/699 (22.3)	236/764 (30.9)	247/908 (27.2)	355/1262 (28.1)	418/1426 (29.3)	428/1617 (26.5)	2139/8093 (26.4)	<0.001
Twin or multiple pregnancy	64/221 (29.0)	127/324 (39.2)	188/415 (45.3)	201/474 (42.4)	259/701 (36.9)	324/765 (42.4)	367/936 (39.2)	488/1286 (37.9)	523/1443 (36.2)	595/1656 (35.9)	3136/8221 (38.1)	<0.001
Cesarean delivery	52/223 (23.3)	47/325 (14.5)	65/414 (15.7)	91/477 (19.1)	123/702 (17.5)	117/767 (15.3)	144/966 (14.9)	232/1290 (18.0)	318/1452 (21.9)	416/1648 (25.2)	1605/8264 (19.4)	<0.001
PROM	64/223 (28.7)	91/323 (28.2)	113/416 (27.2)	149/476 (31.3)	238/700 (34.0)	253/767 (33.0)	314/960 (32.7)	467/1293 (36.1)	479/1453 (33.0)	538/1590 (33.8)	2706/8201 (33.0)	0.002
>18h	37/219 (16.9)	55/322 (17.1)	64/408 (15.7)	85/465 (18.3)	149/687 (21.7)	159/753 (21.1)	198/939 (21.1)	321/1265 (25.4)	323/1431 (22.6)	326/1569 (20.8)	1717/8058 (21.3)	<0.001
>24h	32/219 (14.6)	46/322 (14.3)	63/408 (15.4)	67/465 (14.4)	127/687 (18.5)	144/753 (19.1)	174/939 (18.5)	282/1265 (22.3)	288/1431 (20.1)	272/1569 (17.3)	1495/8058 (18.6)	0.002
Chorioamnionitis	1/223 (0.4)	1/324 (0.3)	4/413 (1.0)	7/475 (1.5)	7/701 (1.0)	6/767 (0.8)	10/938 (1.1)	18/1271 (1.4)	38/1410 (2.7)	67/1588 (4.2)	159/8110 (2.0)	<0.001
Placental abruption / Placenta previa	16/223 (7.2)	27/324 (8.3)	40/413 (9.7)	34/475 (7.2)	54/701 (7.7)	52/767 (6.8)	94/938 (10.0)	98/1271 (7.7)	146/1410 (10.4)	168/1588 (10.6)	729/8110 (9.0)	0.03
HDCP	25/223 (11.2)	29/325 (8.9)	28/416 (6.7)	34/477 (7.1)	48/703 (6.8)	57/767 (7.4)	59/938 (6.3)	111/1271 (8.7)	123/1432 (8.6)	166/1656 (10.0)	680/8208 (8.3)	0.20
GDM	10/223 (4.5)	14/325 (4.3)	30/416 (7.2)	23/477 (4.8)	50/703 (7.1)	103/767 (13.4)	64/938 (6.8)	144/1271 (11.3)	200/1432 (14.0)	247/1656 (14.9)	885/8208 (10.8)	<0.001
Antenatal steroids	54/223 (24.2)	80/322 (24.8)	106/416 (25.5)	137/476 (28.8)	232/700 (33.1)	309/767 (40.3)	461/947 (48.7)	695/1291 (53.8)	889/1450 (61.3)	957/1650 (58.0)	3920/8242 (47.6)	<0.001

© 2021 Zhu Z et al. *JAMA Network Open.*

Abbreviation: BW, birth weight; GA, gestational age; GDM, gestational diabetes mellitus; HDCP, hypertensive disorder complicating pregnancy; PROM, premature rupture of membranes; SD, standard deviation; SGA, small for gestational age.

[a]Data were shown as n/N* (%) unless otherwise indicated. Denominators (N*) varied according to the number of missing data for each variable.

[b]*P* values were determined for trend over the decade using modified Poisson regression or linear regression models, with adjustment for GA, BW, and study site. Differences in GA were only adjusted for BW and study site. *P* value for trend of the proportion of extremely preterm infants in all discharged infants was not adjusted.

[c]Proportion of extremely preterm infants in all discharged infants from NICUs. Numerator only includes extremely preterm infants from hospitals with available data for total number of discharged infants in the corresponding study year.

© 2021 Zhu Z et al. *JAMA Network Open*.

eTable 2. Maternal and Infant Characteristics for Extremely Preterm Births by Region[a]

Characteristics	Northeast (N=375)	North (N=1153)	East (N=3566)	Central (N=1269)	South (N=1061)	Northwest (N=474)	Southwest (N=616)	Total (N=8514)
GA, mean (SD), wk	26.9 (0.9)	26.7 (1.0)	26.8 (1.0)	26.8 (0.9)	26.5 (1.1)	26.7 (1.1)	26.8 (1.0)	26.7 (1.0)
BW, mean (SD), g	1006.3 (183.7)	968.4 (184.8)	971.0 (175.8)	979.0 (172.4)	915.5 (185.7)	974.6 (202.7)	968.0 (170.9)	966.5 (180.5)
SGA	5/374 (1.3)	30/1151 (2.6)	64/3553 (1.8)	18/1265 (1.4)	30/1054 (2.8)	11/470 (2.3)	8/611 (1.3)	166/8478 (2.0)
Male	212/375 (56.5)	772/1153 (67.0)	2148/3566 (60.2)	860/1269 (67.8)	662/1061 (62.4)	277/474 (58.4)	364/616 (59.1)	5295/8514 (62.2)
Fetal distress	25/375 (6.7)	65/1153 (5.6)	165/3527 (4.7)	123/1047 (11.7)	45/1061 (4.2)	40/474 (8.4)	69/605 (11.4)	532/8242 (6.5)
5-min Apgar score≤7	238/287 (82.9)	374/1065 (35.1)	1336/3303 (40.4)	329/923 (35.6)	167/1040 (16.1)	189/447 (42.3)	204/550 (37.1)	2837/7615 (37.3)
Elderly pregnancy	57/359 (15.9)	168/848 (19.8)	672/3445 (19.5)	179/1034 (17.3)	192/996 (19.3)	58/466 (12.4)	82/592 (13.9)	1408/7740 (18.2)
In vitro fertilization	81/352 (23.0)	281/1105 (25.4)	923/3510 (26.3)	310/1018 (30.5)	253/1044 (24.2)	128/466 (27.5)	163/598 (27.3)	2139/8093 (26.4)
Twin or multiple pregnancy	133/375 (35.5)	386/1128 (34.2)	1294/3531 (36.6)	445/1047 (42.5)	401/1061 (37.8)	200/474 (42.2)	277/605 (45.8)	3136/8221 (38.1)
Cesarean delivery	46/375 (12.3)	307/1151 (26.7)	706/3557 (19.8)	202/1043 (19.4)	187/1060 (17.6)	85/473 (18.0)	72/605 (11.9)	1605/8264 (19.4)
PROM	119/375 (31.7)	334/1148 (29.1)	1202/3498 (34.4)	381/1041 (36.6)	294/1061 (27.7)	151/474 (31.9)	225/604 (37.3)	2706/8201 (33.0)
Chorioamnionitis	1/375 (0.3)	16/1147 (1.4)	66/3409 (1.9)	19/1039 (1.8)	22/1061 (2.1)	5/474 (1.1)	30/605 (5.0)	159/8110 (2.0)
Placental abruption / Placenta previa	27/375 (7.2)	114/1147 (9.9)	280/3409 (8.2)	96/1039 (9.2)	96/1061 (9.0)	44/474 (9.3)	72/605 (11.9)	729/8110 (9.0)
HDCP	25/375 (6.7)	164/1153 (14.2)	281/3493 (8.0)	72/1047 (6.9)	67/1061 (6.3)	49/474 (10.3)	22/605 (3.6)	680/8208 (8.3)
GDM	36/375 (9.6)	135/1153 (11.7)	410/3493 (11.7)	112/1047 (10.7)	95/1061 (9.0)	34/474 (7.2)	63/605 (10.4)	885/8208 (10.8)
Antenatal steroids	100/372 （26.9）	451/1153 （39.1）	1691/3547 （47.7）	580/1038 （55.9）	639/1060 （60.3）	195/473 （41.2）	264/599 （44.1）	3920/8242 （47.6）

Abbreviation: BW, birth weight; GA, gestational age; GDM, gestational diabetes mellitus; HDCP, hypertensive disorder complicating pregnancy; PROM, premature rupture of membranes; SD, standard deviation; SGA, small for gestational age.

[a]Data were shown as n/N* (%) unless otherwise indicated. Denominators (N*) varied according to the number of missing data for each variable.

© 2021 Zhu Z et al. *JAMA Network Open.*

eTable 3. Survival and Major Morbidity of Infants Born Extremely Preterm From 2010 to 2019[a]

Variable	2010 (N=241)	2011 (N=347)	2012 (N=439)	2013 (N=537)	2014 (N=752)	2015 (N=828)	2016 (N=968)	2017 (N=1293)	2018 (N=1453)	2019 (N=1656)	Total (N=8514)	*P* value[c]
Survival	136/241 (56.4)	186/347 (53.6)	245/439 (55.8)	294/537 (54.7)	447/752 (59.4)	484/828 (58.5)	584/968 (60.3)	825/1293 (63.8)	990/1453 (68.1)	1111/1656 (67.1)	5302/8514 (62.3)	<0.001
GA 21-23 wk[b]	0/0	1/2 (50.0)	0/9	0/2	0/3	3/18 (16.7)	2/17 (11.8)	1/5 (20.0)	6/17 (35.3)	1/23 (4.3)	14/96 (14.6)	0.21
GA 24 wk	5/11 (45.5)	3/18 (16.7)	6/18 (33.3)	3/16 (18.8)	9/34 (26.5)	6/25 (24.0)	18/46 (39.1)	25/69 (36.2)	38/85 (44.7)	31/86 (36.0)	144/408 (35.3)	0.002
GA 25 wk	10/22 (45.5)	11/26 (42.3)	15/39 (38.5)	21/44 (47.7)	37/85 (43.5)	42/103 (40.8)	65/122 (53.3)	82/163 (50.3)	85/169 (50.3)	112/214 (52.3)	480/987 (48.6)	<0.001
GA 26 wk	42/69 (60.9)	35/85 (41.2)	59/125 (47.2)	78/141 (55.3)	117/196 (59.7)	122/228 (53.5)	132/231 (57.1)	234/359 (65.2)	293/442 (66.3)	311/455 (68.4)	1423/2331 (61.0)	<0.001
GA 27 wk	79/139 (56.8)	136/216 (63.0)	165/248 (66.5)	192/334 (57.5)	284/434 (65.4)	311/454 (68.5)	367/552 (66.5)	483/697 (69.3)	568/740 (76.8)	656/878 (74.7)	3241/4692 (69.1)	<0.001
Active treatment	187/241 (77.6)	255/347 (73.5)	326/439 (74.3)	388/537 (72.3)	546/752 (72.6)	605/828 (73.1)	684/968 (70.7)	939/1293 (72.6)	1095/1453 (75.4)	1252/1656 (75.6)	6277/8514 (73.7)	<0.001
Death despite active treatment	51/241 (21.2)	69/347 (19.9)	81/439 (18.5)	94/537 (17.5)	99/752 (13.2)	121/828 (14.6)	100/968 (10.3)	114/1293 (8.8)	105/1453 (7.2)	141/1656 (8.5)	975/8514 (11.5)	<0.001
Death after withdrawing treatment	54/241 (22.4)	92/347 (26.5)	113/439 (25.7)	149/537 (27.7)	206/752 (27.4)	223/828 (26.9)	284/968 (29.3)	354/1293 (27.4)	358/1453 (24.6)	404/1656 (24.4)	2237/8514 (26.3)	<0.001
Postnatal days until death (N=3073)												
<1 days	11/95 (11.6)	23/150 (15.3)	15/186 (8.1)	29/211 (13.7)	38/284 (13.4)	50/319 (15.7)	51/378 (13.5)	54/451 (12.0)	58/454 (12.8)	85/545 (15.6)	414/3073 (13.5)	0.09

© 2021 Zhu Z et al. *JAMA Network Open.*

Variable	2010 (N=241)	2011 (N=347)	2012 (N=439)	2013 (N=537)	2014 (N=752)	2015 (N=828)	2016 (N=968)	2017 (N=1293)	2018 (N=1453)	2019 (N=1656)	Total (N=8514)	*P* value[c]
1-3 days	42/95 (44.2)	55/150 (36.7)	78/186 (41.9)	91/211 (43.1)	114/284 (40.1)	123/319 (38.6)	119/378 (31.5)	161/451 (35.7)	155/454 (34.1)	165/545 (30.3)	1103/3073 (35.9)	<0.001
4-7 days	14/95 (14.7)	22/150 (14.7)	21/186 (11.3)	32/211 (15.2)	37/284 (13.0)	37/319 (11.6)	64/378 (16.9)	68/451 (15.1)	57/454 (12.6)	75/545 (13.8)	427/3073 (13.9)	0.44
8-28 days	16/95 (16.8)	33/150 (22.0)	52/186 (28.0)	28/211 (13.3)	57/284 (20.1)	73/319 (22.9)	77/378 (20.4)	90/451 (20.0)	97/454 (21.4)	116/545 (21.3)	639/3073 (20.8)	0.11
>28 days	12/95 (12.6)	17/150 (11.3)	20/186 (10.8)	31/211 (14.7)	38/284 (13.4)	36/319 (11.3)	67/378 (17.7)	78/451 (17.3)	87/454 (19.2)	104/545 (19.1)	490/3073 (15.9)	<0.001
Length of stay for survivors, median (IQR), d	58 (59)	62 (45)	67 (40)	62 (42)	63 (40)	70 (29)	67.5 (28)	69 (30)	69 (31)	72 (30)	68 (32)	<0.001
Corrected GA at discharge for survivors, mean (SD), wk	36.3 (6.0)	36.7 (4.8)	37.2 (5.2)	36.4 (4.5)	36.6 (4.8)	37.2 (4.0)	37.5 (3.9)	37.7 (4.0)	37.8 (3.9)	38.5 (4.4)	37.6 (4.3)	<0.001
Cranial imaging	222/241 (92.1)	314/347 (90.5)	411/439 (93.6)	464/537 (86.4)	693/752 (92.2)	738/828 (89.1)	815/968 (84.2)	1121/1293 (86.7)	1296/1453 (89.2)	1492/1656 (90.1)	7566/8514 (88.9)	0.06
ROP screening	93/223 (41.7)	136/325 (41.8)	184/416 (44.2)	220/477 (46.1)	352/703 (50.1)	377/767 (49.2)	570/968 (58.9)	788/1293 (60.9)	907/1453 (62.4)	1047/1656 (63.2)	4674/8281 (56.4)	<0.001
Major morbidities												
BPD[d]	64/115 (55.7)	81/167 (48.5)	127/222 (57.2)	169/262 (64.5)	292/395 (73.9)	332/454 (73.1)	453/614 (73.8)	617/806 (76.6)	746/950 (78.5)	954/1194 (79.9)	3835/5179 (74.0)	<0.001
IVH (Grade III-IV)[e]	14/219 (6.4)	27/311 (8.7)	57/397 (14.4)	51/457 (11.2)	70/683 (10.2)	108/723 (14.9)	129/800 (16.1)	159/1079 (14.7)	148/1249 (11.8)	248/1444 (17.2)	1011/7362 (13.7)	<0.001

© 2021 Zhu Z et al. *JAMA Network Open.*

Variable	2010 (N=241)	2011 (N=347)	2012 (N=439)	2013 (N=537)	2014 (N=752)	2015 (N=828)	2016 (N=968)	2017 (N=1293)	2018 (N=1453)	2019 (N=1656)	Total (N=8514)	*P* value[c]
WMI[e]	29/222 (13.1)	45/314 (14.3)	53/411 (12.9)	33/464 (7.1)	91/693 (13.1)	126/738 (17.1)	141/815 (17.3)	214/1121 (19.1)	231/1296 (17.8)	299/1492 (20.0)	1262/7566 (16.7)	0.001
NEC (Stage II-III)	8/222 (3.6)	11/324 (3.4)	17/415 (4.1)	12/476 (2.5)	43/703 (6.1)	64/758 (8.4)	67/959 (7.0)	77/1279 (6.0)	96/1438 (6.7)	136/1572 (8.7)	531/8146 (6.5)	<0.001
Sepsis	55/223 (24.7)	76/325 (23.4)	111/416 (26.7)	140/477 (29.4)	203/703 (28.9)	266/767 (34.7)	353/948 (37.2)	484/1291 (37.5)	546/1453 (37.6)	762/1656 (46.0)	2996/8259 (36.3)	<0.001
Severe ROP[f]	18/93 (19.4)	15/133 (11.3)	31/183 (16.9)	17/217 (7.8)	77/343 (22.4)	84/368 (22.8)	119/547 (21.8)	135/752 (18.0)	152/871 (17.5)	200/1011 (19.8)	848/4518 (18.8)	0.28
Any major morbidity	116/223 (52.0)	172/325 (52.9)	250/416 (60.1)	270/477 (56.6)	443/703 (63.0)	547/767 (71.3)	716/968 (74.0)	986/1293 (76.3)	1136/1453 (78.2)	1363/1656 (82.3)	5999/8281 (72.4)	<0.001
GA 21-23 wk[b]	0/0	1/2 (50.0)	1/9 (11.1)	1/2 (50.0)	0/3	9/18 (50.0)	9/17 (52.9)	3/5 (60.0)	12/17 (70.6)	11/23 (47.8)	47/96 (49.0)	0.21
GA 24 wk	4/10 (40.0)	11/17 (64.7)	7/18 (38.9)	5/15 (33.3)	17/32 (53.1)	15/24 (62.5)	39/46 (84.8)	51/69 (73.9)	55/85 (64.7)	57/86 (66.3)	261/402 (64.9)	0.002
GA 25 wk	13/22 (59.1)	14/23 (60.9)	22/37 (59.5)	19/40 (47.5)	46/83 (55.4)	60/96 (62.5)	85/122 (69.7)	123/163 (75.5)	121/169 (71.6)	172/214 (80.4)	675/969 (69.7)	<0.001
GA 26 wk	34/65 (52.3)	47/80 (58.8)	78/118 (66.1)	69/124 (55.6)	113/180 (62.8)	148/208 (71.2)	181/231 (78.4)	274/359 (76.3)	362/442 (81.9)	384/455 (84.4)	1690/2262 (74.7)	<0.001
GA 27 wk	65/126 (51.6)	99/203 (48.8)	142/234 (60.7)	176/296 (59.5)	267/405 (65.9)	315/421 (74.8)	402/552 (72.8)	535/697 (76.8)	586/740 (79.2)	739/878 (84.2)	3326/4552 (73.1)	<0.001
Survival without major morbidity[g]	48/223 (21.5)	61/325 (18.8)	57/416 (13.7)	79/477 (16.6)	100/703 (14.2)	59/767 (7.7)	76/968 (7.9)	98/1293 (7.6)	110/1453 (7.6)	92/1656 (5.6)	780/8281 (9.4)	<0.001

Abbreviation: BPD, bronchopulmonary dysplasia; GA, gestational age; IQR, interquartile range; IVH, intraventricular hemorrhage; NEC, necrotizing enterocolitis; ROP, retinopathy of prematurity; SD, standard deviation; WMI, white matter injury.

© 2021 Zhu Z et al. *JAMA Network Open.*

[a]Data were shown as n/N* (%) unless otherwise indicated. Denominators (N*) varied according to the number of missing data for each variable.

[b]Data were combined to present into one category because of the small number for analysis, including 4 infants born at 21 weeks, 21 infants born at 22 weeks, and 71 infants at 23 weeks.

[c]*P* values were determined for trend over the decade using modified Poisson regression or linear regression models, with adjustment for GA, BW, and study site. *P* values for the survival trend and trend of major morbidity stratified by GA were only adjusted for BW and study site. Differences in length of stay were analyzed by Spearman rank correlation test.

[d]Proportions among infants who survived more than 28 days of life. Denominator included survivors with obtainable postnatal days until discharge over 28 days (n=4700) and those who died after 28 postnatal days (n=490). Diagnosis of BPD was unknown for 11 infants.

[e]Proportions among infants who underwent cranial imaging (cranial sonography, magnetic resonance imaging, or computed tomography) [n=7566]. IVH grade was unknown for 204 infants.

[f]Proportions among infants who received ROP examination (n=4674). The stage or treatments of ROP were not available for 156 infants.

[g]Proportions among the study population. Diagnosis of major morbidity was unknown for 233 infants.

© 2021 Zhu Z et al. *JAMA Network Open*.

eTable 4. Survival and Major Morbidity of Infants Born Extremely Preterm by Region

Characteristics	Northeast (N=375)	North (N=1153)	East (N=3566)	Central (N=1269)	South (N=1061)	Northwest (N=474)	Southwest (N=616)	Total (N=8514)
Survival	164/375 (43.7)	887/1153 (76.9)	2279/3566 (63.9)	781/1269 (61.5)	729/1061 (68.7)	188/474 (39.7)	274/616 (44.5)	5302/8514 (62.3)
Active treatment	236/375 (62.9)	977/1153 (84.7)	2677/3566 (75.1)	964/1269 (76.0)	852/1061 (80.3)	238/474 (50.2)	333/616 (54.1)	6277/8514 (73.7)
Death despite active treatment	72/375 (19.2)	90/1153 (7.8)	398/3566 (11.2)	183/1269 (14.4)	123/1061 (11.6)	50/474 (10.5)	59/616 (9.6)	975/8514 (11.5)
Death after withdrawing treatment	139/375 (37.1)	176/1153 (15.3)	889/3566 (24.9)	305/1269 (24.0)	209/1061 (19.7)	236/474 (49.8)	283/616 (45.9)	2237/8514 (26.3)
Length of stay for survivors, median (IQR), d	64 (29)	60 (46)	69 (31)	67 (35)	75 (29)	66 (29)	64 (26)	68 (32)
Corrected GA at discharge for survivors, mean (SD), wk	36.5 (3.8)	37.1 (5.7)	37.7 (3.8)	38 (4.8)	38.1 (4.2)	37.3 (3.6)	36.6 (3.7)	37.6 (4.3)
Cranial imaging	298/375 (79.5)	1109/1153 (96.2)	3307/3566 (92.7)	939/1269 (74.0)	1014/1061 (95.6)	415/474 (87.6)	484/616 (78.6)	7566/8514 (88.9)
ROP screening	153/375 (40.8)	790/1153 (68.5)	2099/3566 (58.9)	611/1047 (58.4)	567/1061 (53.4)	194/474 (40.9)	260/605 (43.0)	4674/8281 (56.4)
Major morbidities								
BPD[b]	108/172 (62.8)	381/651 (58.5)	1826/2416 (75.6)	541/692 (78.2)	595/727 (81.8)	156/218 (71.6)	228/303 (75.2)	3835/5179 (74.0)
IVH (Grade III-IV)[c]	69/298 (23.2)	122/1095 (11.1)	375/3183 (11.8)	237/922 (25.7)	111/1002 (11.1)	50/385 (13.0)	47/477 (9.9)	1011/7362 (13.7)

© 2021 Zhu Z et al. *JAMA Network Open.*

Characteristics	Northeast (N=375)	North (N=1153)	East (N=3566)	Central (N=1269)	South (N=1061)	Northwest (N=474)	Southwest (N=616)	Total (N=8514)
WMI[c]	36/298 (12.1)	185/1109 (16.7)	595/3307 (18.0)	230/939 (24.5)	63/1014 (6.2)	55/415 (13.3)	98/484 (20.2)	1262/7566 (16.7)
NEC (Stage II-III)	21/372 (5.6)	68/1149 (5.9)	234/3460 (6.8)	107/1037 (10.3)	37/1061 (3.5)	38/471 (8.1)	26/596 (4.4)	531/8146 (6.5)
Sepsis	133/375 (35.5)	300/1153 (26.0)	1400/3544 (39.5)	525/1047 (50.1)	247/1061 (23.3)	151/474 (31.9)	240/605 (39.7)	2996/8259 (36.3)
Severe ROP[d]	31/152 (20.4)	182/785 (23.2)	291/2035 (14.3)	135/572 (23.6)	135/552 (24.5)	30/177 (16.9)	44/245 (18.0)	848/4518 (18.8)
Any major morbidity	249/375 (66.4)	803/1153 (69.6)	2662/3566 (74.6)	854/1047 (81.6)	756/1061 (71.3)	292/474 (61.6)	383/605 (63.3)	5999/8281 (72.4)
Survival without major morbidity[e]	22/375 (5.9)	230/1153 (19.9)	291/3566 (8.2)	62/1047 (5.9)	110/1061 (10.4)	30/474 (6.3)	35/605 (5.8)	780/8281 (9.4)

Abbreviation: BPD, bronchopulmonary dysplasia; GA, gestational age; IQR, interquartile range; IVH, intraventricular hemorrhage; NEC, necrotizing enterocolitis; ROP, retinopathy of prematurity; SD, standard deviation; WMI, white matter injury.

[a]Data were shown as n/N* (%) unless otherwise indicated. Denominators (N*) varied according to the number of missing data for each variable.

[b]Proportions among infants who survived more than 28 days of life. Denominator included survivors with obtainable postnatal days until discharge over 28 days (n=4700) and those who died after 28 postnatal days (n=490). Diagnosis of BPD was unknown for 11 infants.

[c]Proportions among infants who underwent cranial imaging (cranial sonography, magnetic resonance imaging, or computed tomography) [n=7566]. IVH grade was unknown for 204 infants.

[d]Proportions among infants who received ROP examination (n=4674). The stage or treatments of ROP were not available for 156 infants.

[e]Proportions among the study population. Diagnosis of major morbidity was unknown for 233 infants.

© 2021 Zhu Z et al. *JAMA Network Open*.

eTable 5. Survival and Major Morbidity of Infants Born Extremely Preterm by Region From 2010 to 2019[a]

Variable	2010 (N=241)	2011 (N=347)	2012 (N=439)	2013 (N=537)	2014 (N=752)	2015 (N=828)	2016 (N=968)	2017 (N=1293)	2018 (N=1453)	2019 (N=1656)	Total (N=8514)	*P* value[b]
Survival												
Northeast	2/12 (16.7)	5/24 (20.8)	7/25 (28.0)	12/39 (30.8)	26/60 (43.3)	29/70 (41.4)	19/41 (46.3)	22/44 (50.0)	24/32 (75.0)	18/28 (64.3)	164/375 (43.7)	<0.001
North	45/57 (78.9)	61/73 (83.6)	71/89 (79.8)	75/85 (88.2)	122/145 (84.1)	55/74 (74.3)	51/88 (58.0)	99/141 (70.2)	148/186 (79.6)	160/215 (74.4)	887/1153 (76.9)	0.11
East	48/95 (50.5)	53/120 (44.2)	78/163 (47.9)	107/202 (53.0)	175/311 (56.3)	221/356 (62.1)	285/432 (66.0)	397/572 (69.4)	463/659 (70.3)	452/656 (68.9)	2279/3566 (63.9)	<0.001
Central	18/33 (54.5)	35/56 (62.5)	33/49 (67.3)	42/94 (44.7)	52/92 (56.5)	75/122 (61.5)	120/190 (63.2)	106/192 (55.2)	123/192 (64.1)	177/249 (71.1)	781/1269 (61.5)	<0.001
South	15/23 (65.2)	26/53 (49.1)	41/74 (55.4)	44/63 (69.8)	54/81 (66.7)	71/112 (63.4)	71/105 (67.6)	118/166 (71.1)	152/202 (75.2)	137/182 (75.3)	729/1061 (68.7)	<0.001
Northwest	4/9 (44.4)	3/8 (37.5)	7/18 (38.9)	6/19 (31.6)	10/32 (31.3)	14/36 (38.9)	17/58 (29.3)	39/90 (43.3)	27/70 (38.6)	61/134 (45.5)	188/474 (39.7)	0.05
Southwest	4/12 (33.3)	3/13 (23.1)	8/21 (38.1)	8/35 (22.9)	8/31 (25.8)	19/58 (32.8)	21/54 (38.9)	44/88 (50.0)	53/112 (47.3)	106/192 (55.2)	274/616 (44.5)	<0.001
Any major morbidity[c]												
Northeast	5/12 (41.7)	11/24 (45.8)	17/25 (68.0)	21/39 (53.8)	46/60 (76.7)	50/70 (71.4)	30/41 (73.2)	23/44 (52.3)	25/32 (78.1)	21/28 (75.0)	249/375 (66.4)	0.03
North	25/57 (43.9)	33/73 (45.2)	52/89 (58.4)	40/85 (47.1)	76/145 (52.4)	49/74 (66.2)	70/88 (79.5)	116/141 (82.3)	161/186 (86.6)	181/215 (84.2)	803/1153 (69.6)	<0.001
East	57/95 (60.0)	75/120 (62.5)	101/163 (62.0)	120/202 (59.4)	217/311 (69.8)	268/356 (75.3)	311/432 (72.0)	440/572 (76.9)	518/659 (78.6)	555/656 (84.6)	2662/3566 (74.6)	<0.001

© 2021 Zhu Z et al. *JAMA Network Open.*

Variable	2010 (N=241)	2011 (N=347)	2012 (N=439)	2013 (N=537)	2014 (N=752)	2015 (N=828)	2016 (N=968)	2017 (N=1293)	2018 (N=1453)	2019 (N=1656)	Total (N=8514)	*P* value[b]
Central	8/15 (53.3)	21/34 (61.8)	16/26 (61.5)	23/37 (62.2)	31/46 (67.4)	52/66 (78.8)	166/190 (87.4)	154/192 (80.2)	162/192 (84.4)	221/249 (88.8)	854/1047 (81.6)	<0.001
South	15/23 (65.2)	27/53 (50.9)	52/74 (70.3)	43/63 (68.3)	55/81 (67.9)	77/112 (68.8)	78/105 (74.3)	122/166 (73.5)	140/202 (69.3)	147/182 (80.8)	756/1061 (71.3)	0.001
Northwest	4/9 (44.4)	5/8 (62.5)	8/18 (44.4)	11/19 (57.9)	13/32 (40.6)	23/36 (63.9)	31/58 (53.4)	62/90 (68.9)	48/70 (68.6)	87/134 (64.9)	292/474 (61.6)	0.003
Southwest	116/223 (52.0)	172/325 (52.9)	250/416 (60.1)	270/477 (56.6)	443/703 (63.0)	547/767 (71.3)	716/968 (74.0)	986/1293 (76.3)	1136/1453 (78.2)	1363/1656 (82.3)	5999/8281 (72.4)	<0.001

[a]Data were shown as n/N* (%) unless otherwise indicated. Denominators (N*) varied according to the number of missing data for each variable.

[b]*P* values were determined for trend over the decade using modified Poisson regression models, with adjustment for GA and BW.

[c]Diagnosis of major morbidity was unknown for 233 infants.

© 2021 Zhu Z et al. *JAMA Network Open*.

eTable 6. Perinatal and Maternal Characteristics for Infants Who Survived vs Did Not Survive[a]

	**Survivors (N=5302)**	**Non-survivors (N=3212)**	***P* value[b]**
GA, mean (SD), wk	26.9 (0.8)	26.5 (1.1)	<0.001
BW, mean (SD), g	998.1 (169.7)	914.1 (185.7)	<0.001
SGA	61/5290 (1.2)	105/3188 (3.3)	<0.001
Male	3340/5302 (63.0)	1955/3212 (60.9)	0.05
Fetal distress	286/5137 (5.6)	246/3105 (7.9)	<0.001
5-min Apgar score ≤ 7	1474/4811 (30.6)	1363/2804 (48.6)	<0.001
Elderly pregnancy	967/4824 (20.0)	441/2916 (15.1)	<0.001
In vitro fertilization	1389/5073 (27.4)	750/3020 (24.8)	0.01
Twin or multiple pregnancy, n (%)	1816/5135 (35.4)	1320/3086 (42.8)	<0.001
Cesarean delivery	1084/5167 (21.0)	521/3097 (16.8)	<0.001
PROM	1785/5127 (34.8)	921/3074 (30.0)	<0.001
Chorioamnionitis	100/5048 (2.0)	59/3062 (1.9)	0.87
Placental abruption / Placenta previa	439/5048 (8.7)	290/3062 (9.5)	0.24
HDCP	436/5119 (8.5)	244/3089 (7.9)	0.33
GDM	588/5119 (11.5)	297/3089 (9.6)	0.008
Antenatal steroids	2728/5154 (52.9)	1192/3088 (38.6)	<0.001

[a]Data were shown as n/N* (%) unless otherwise indicated. Denominators (N*) varied according to the number of missing data for each variable.

[b]Group differences were explored among the total cohort using t-test for continuous variables and chi-square test for dichotomous variables without adjustment.

© 2021 Zhu Z et al. *JAMA Network Open.*

eTable 7. Multivariable Regression Analysis of Perinatal Risk Factors for Survival in Total Study Group

Variable	Crude model		Adjusted model[a]	
	**RR (95% CI)**	***P* value**	**aRR (95% CI)**	***P* value**
GA (wk)	1.120 (1.088-1.152)	<0.001	1.139 (1.108-1.170)	<0.001
BW group (100g intervals)	1.060 (1.047-1.073)	<0.001	1.075 (1.062-1.087)	<0.001
SGA	0.752 (0.600-0.944)	0.01	0.753 (0.610-0.929)	0.008
Male	0.994 (0.959-1.031)	0.76	1.002 (0.968-1.037)	0.90
Fetal distress	0.869 (0.801-0.943)	0.001	0.950 (0.877-1.030)	0.22
5-min Apgar score≤7	0.816 (0.783-0.851)	<0.001	0.877 (0.840-0.915)	<0.001
Elderly pregnancy	1.098 (1.054-1.143)	<0.001	1.071 (1.030-1.113)	<0.001
In vitro fertilization	1.126 (1.078-1.177)	<0.001	1.095 (1.049-1.143)	<0.001
Twin or multiple pregnancy	0.867 (0.829-0.905)	<0.001	0.894 (0.857-0.931)	<0.001
Cesarean delivery	1.075 (1.029-1.123)	0.001	1.005 (0.963-1.048)	0.823
PROM	1.037 (1.000-1.076)	0.05	1.046 (1.010-1.083)	0.01
Chorioamnionitis	0.984 (0.874-1.108)	0.79	1.050 (0.934-1.181)	0.41
Placental abruption / Placenta previa	0.932 (0.873-0.995)	0.03	0.961 (0.904-1.022)	0.20
HDCP	1.024 (0.959-1.094)	0.48	1.066 (1.001-1.134)	0.05
GDM	0.967 (0.917-1.019)	0.21	0.934 (0.888-0.983)	0.009
Antenatal steroids	1.192 (1.149-1.237)	<0.001	1.097 (1.056-1.139)	<0.001

Abbreviation: BW, birth weight; CI, confidence interval; GA, gestational age; GDM, gestational diabetes mellitus; HDCP, hypertensive disorder complicating pregnancy; PROM, premature rupture of membranes; RR, risk ratio; SGA, small for gestational age.

[a]Analysis was performed with adjustment for study site and discharge year.

© 2021 Zhu Z et al. *JAMA Network Open.*
